# Influence of ultrasound machine settings on quantitative measures derived from spatial frequency analysis of muscle tissue

**DOI:** 10.1186/s12891-023-06790-3

**Published:** 2023-08-22

**Authors:** Scott K. Crawford, Stephanie A. Kliethermes, Bryan C. Heiderscheit, Greg R. Bashford

**Affiliations:** 1 Department of Kinesiology, University of Wisconsin-Madison, 1300 University Ave, Madison, WI 53706 USA; 2 Department of Orthopedics & Rehabilitation, University of Wisconsin-Madison, Madison, WI USA; 3 Badger Athletic Performance Program, University of Wisconsin-Madison, Madison, WI USA; 4 Department of Biomedical Engineering, University of Wisconsin-Madison, Madison, WI USA; 5 Department of Biological Systems Engineering, University of Nebraska, Lincoln, NE USA

**Keywords:** Ultrasonography, Hamstring muscles, Spatial frequency, Musculoskeletal, Imaging

## Abstract

**Background:**

Ultrasound is a powerful tool for diagnostic purposes and provides insight into both normal and pathologic tissue structure. Spatial frequency analysis (SFA) methods characterize musculoskeletal tissue organization from ultrasound images. Both sonographers in clinical imaging and researchers may alter a minimized range of ultrasound settings to optimize image quality, and it is important to know how these small adjustments of these settings affect SFA parameters. The purpose of this study was to investigate the effects of making small adjustments in a typical default ultrasound machine setting on extracted spatial frequency parameters (peak spatial frequency radius (PSFR), Mmax, Mmax%, and Sum) in the biceps femoris muscle.

**Methods:**

Longitudinal B-mode images were collected from the biceps femoris muscle in 36 participants. The window depth, foci locations, and gain were systematically adjusted consistent with clinical imaging procedures for a total of 27 images per participant. Images were analyzed by identifying a region of interest (ROI) in the middle portion of the muscle belly in a template image and using a normalized two-dimensional cross-correlation technique between the template image and subsequent images. The ROI was analyzed in the frequency domain using conventional SFA methods. Separate linear mixed effects models were run for each extracted parameter.

**Results:**

PSFR was affected by modifications in focus location only (p < 0.001) with differences noted between all locations. Mmax% was influenced by the interaction of gain and focus location (p < 0.001) but was also independently affected by increasing window depth (p < 0.001). Both Mmax and Sum parameters were sensitive to small changes in machine settings with the interaction of focus location and window depth (p < 0.001 for both parameters) as well as window depth and gain (p < 0.001 for both) influencing the extracted values.

**Conclusions:**

Frequently adjusted imaging settings influence some SFA statistics. PSFR and Mmax% appear to be most robust to small changes in image settings, making them best suited for comparison across individuals and between studies, which is appealing for the clinical utility of the SFA method.

**Supplementary Information:**

The online version contains supplementary material available at 10.1186/s12891-023-06790-3.

## Introduction

Quantitative ultrasound methods have been used to characterize musculotendinous tissue structure in both healthy and pathologic conditions [1–4]. Spatial frequency analysis (SFA) is one such method originally developed to assess intra-tendinous tissue structure [5]. SFA analyzes tissue structure by extracting parameters from the spatial spectrum of multiple sub-regions (“kernels”) of a region of interest and compares these parameters across different subject populations. Previous studies using SFA have successfully discriminated between healthy and tendinopathic tendons [5, 6] and shown a relationship between SFA parameters, indirect tendon stiffness, and elastic modulus in degenerated Achilles tendons [7]. More recently, investigations have adapted SFA methods in the hamstrings muscle group in both healthy individuals [8, 9] and following an acute hamstring strain injury [10].

Studies using SFA in both tendon and muscle research have kept image acquisition settings constant for each individual within the investigation [6, 8, 9, 11–13]. This provides a level of standardization in image acquisition protocols, thereby minimizing possible variability in SFA parameters. However, maintaining universal ultrasound system settings may have limited application between patients, studies, or different ultrasound machines [14].

Several machine settings may be adjusted during image acquisition to optimize image quality in musculoskeletal applications. Typically, sonographers start from a fixed “preset” which represents the image settings most likely to be useful for the anatomy chosen. From there, sonographers typically make small adjustments around the preset. For example, the scanning depth may be adjusted to visualize the anatomical region of interest without wasting screen space deep to the structure(s) of interest [15]. The focal zone is also adjusted to correspond to the location of the target structure(s) within the image, which is performed to maximize the lateral resolution at this location. Finally, the overall gain is adjusted to provide desired brightness of target structures, although the preferred brightness level is subjective [15]. The selection of these settings may also be influenced by subcutaneous adipose thickness, which is known to impact image quality [16–19]. Although the preset provides general settings useful for clinical imaging, altering system settings—usually in combination with each other and as minor variations around the initial preset—provides sonographers and clinicians greater clarity in visualizing a region within the target tissue for determining a clinical diagnosis.

A broader range of ultrasound settings is typically used to visualize lower extremity musculature compared to the superficial Achilles and patellar tendons [10, 13, 20], yet it is unknown how minor changes in ultrasound settings influence the value of SFA parameters. Considering that these parameters relate to the physiological, fascicular organization of the muscle [9] – with the peak spatial frequency radius (PSFR) characterizing the most dominant spacing between fascicles, Mmax corresponding to the strength of the most prominent fascicular banded pattern, Sum corresponding to image brightness, and Mmax% relating to the most prominent fascicle pattern relative to the speckle background – it is pertinent to determine if parameters are influenced by modifying machine settings, and if so, to what extent.

Therefore, the purpose of this study was to investigate the effects of making small changes around preset values of ultrasound machine settings typically modified in clinical imaging (imaging depth, focus location, and gain) on extracted spatial frequency parameters within the lower extremity—specifically the biceps femoris muscle of the hamstrings group—to determine the clinical utility of SFA methods in muscle. This study builds upon our previous investigations in the biceps femoris muscle [8–10], which is of particular interest in sports medicine due to the high rate of injury [21, 22]. It is important to clarify the scope of the present study is not to determine the effect of making wide changes on parameters or to compare parameter settings between ultrasound machines since such differences are not typically made in a clinical setting. Instead, the scope of the study is limited to a sonographer’s typical experience – in particular, the usual practice of starting from an anatomically-targeted preset which has been optimized by a machine manufacturer. Within a subset of the originally proposed parameters [5], we hypothesized that the peak spatial frequency radius (PSFR) and Mmax% parameters would be less sensitive to changes in machine settings due to their characterization of fascicular pattern, while Mmax and Sum parameters would be more sensitive to changes in machine settings due to their dependence on image brightness.

## Materials and methods

### Participants and recruitment

A total of 40 participants were recruited through flyers and by direct email solicitation to University of Wisconsin-Madison running and cycling clubs and local community running clubs. Inclusion criteria were 18–35 years of age, self-reporting of 90 min per week of vigorous-intensity aerobic activity [23], a normal (18.5–24.9 kg/m^2^) body mass index (BMI), no history of lower extremity surgery, and not currently pregnant. The study and all procedures were approved by the University of Wisconsin-Madison’s Health Sciences Institutional Review Board, and all participants provided informed written consent prior to commencing any study procedures.

### Study design and image acquisition

The cross-sectional study design consisted of a single testing session. Each participant was positioned prone on an exam table in a relaxed state with their hips and knees in a neutral position and their feet suspended off the end of the exam table. Ultrasound B-mode images were collected from the biceps femoris muscle on the dominant limb for each participant. Limb dominance was determined by the participant indicating which leg they would use to kick a ball as far as they could. Prior to image acquisition, the total thigh length was measured from the ischial tuberosity to the midline between the femoral condyles. A skin mark was placed to the mid-belly location, which was defined as 50% of the measured thigh length [24–26].

**Fig. 1:**
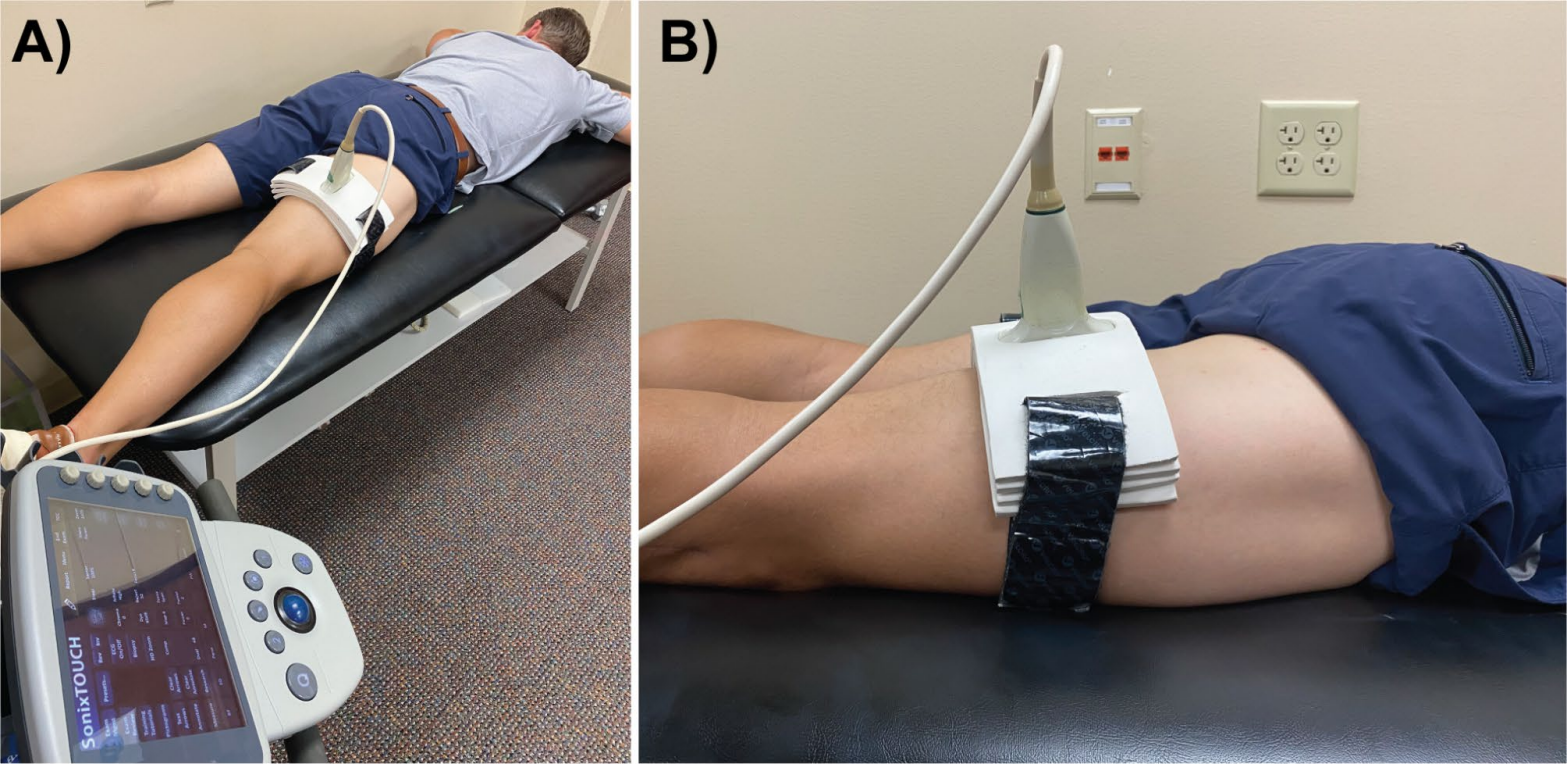
Experimental setup. **A**) The transducer was placed in the fixation mold at the mid-belly of the biceps femoris muscle. **B**) Side view of the transducer in the fixation mold

The same researcher (S.K.C) acquired all images using the same machine (SonixTouch Q+, Ultrasonix Medical Corporation, Richmond, BC, Canada) and linear array transducer (6 cm aperture, L14-5 W/60, Ultrasonix Medical Corporation, Richmond, BC, Canada). Preliminary transverse scans were performed to determine correct positioning for visualizing the biceps femoris muscle. Longitudinal views were then obtained by manipulating the orientation of the transducer to optimize the visualization of the fascicles with parallel orientation of the superficial and intramuscular aponeuroses [27]. The transducer was then placed in a fixation mold to minimize transducer movement and rotation between subsequent image captures. Care was taken when securing the transducer and mold such that minimal pressure was applied to the thigh (Fig. 1). Once the mold was fixed in place, the participant quietly rested for 5 min prior to image acquisition.

### Systematic adjustments of ultrasound setting levels

**Fig. 2:**
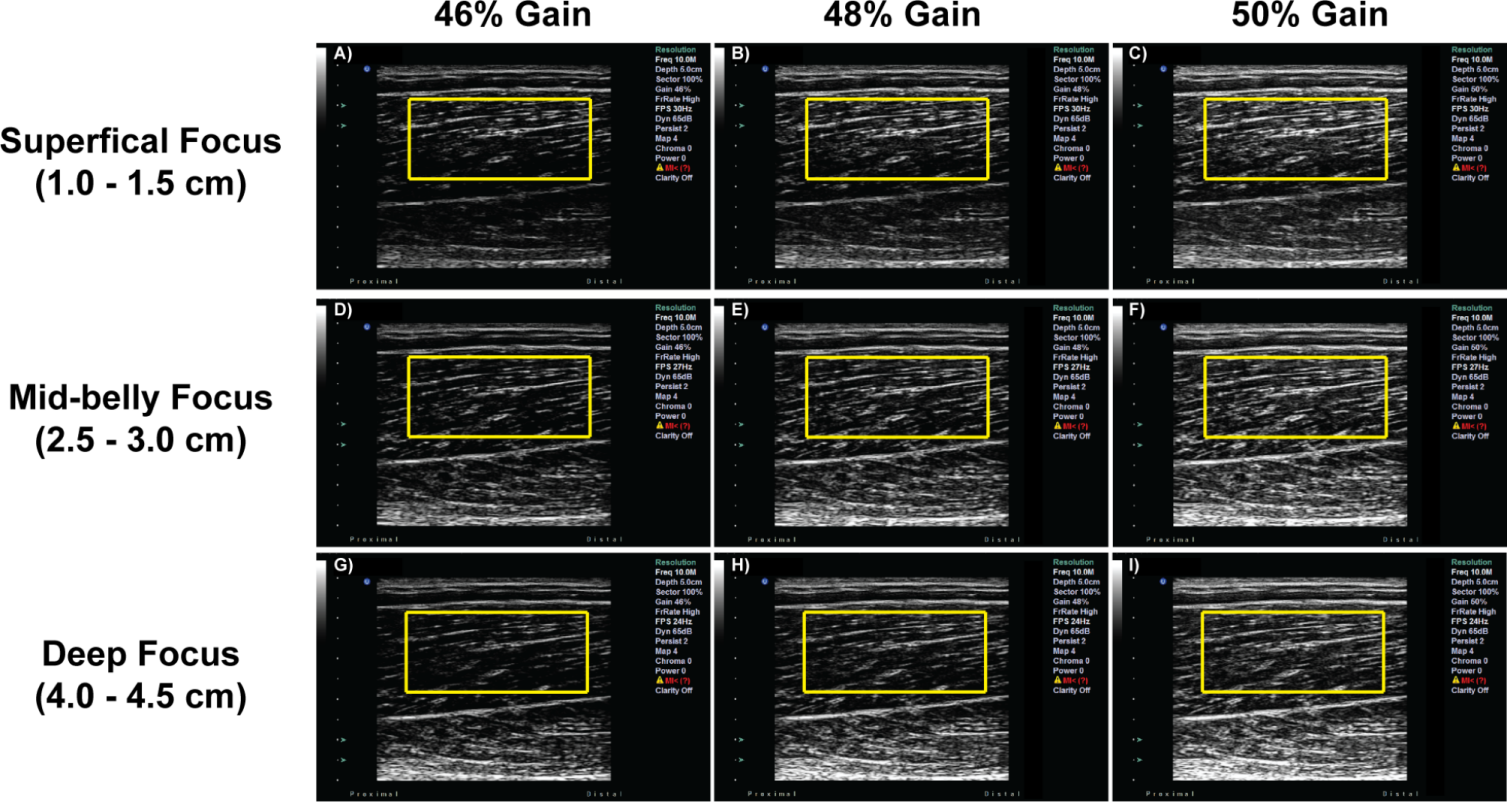
Representative B-mode images of 5 cm window depth from one participant. The rows correspond to the superficial (top), mid-belly (middle), and deep (bottom) focus locations. The columns correspond to 46% (left), 48% (middle), and 50% (right) gain settings. The yellow boxes correspond to the parent ROI of interest from which SFA parameters were extracted. The ROI was drawn using one representative image (for example, image E) and a normalized two-dimensional cross-correlation technique was used to position the parent ROI for each image (**A**-**I**) for subsequent analysis

The “Lower Extremity” preset was used with a transducer frequency of 10 MHz, dynamic range of 65 dB, and dual foci with 0.5 cm spacing between foci. The imaging window depth, focus location, and gain were systematically changed to acquire longitudinal B-mode images with three levels around the preset specified for each machine setting. Previous ultrasound imaging studies investigating architectural measures of the biceps femoris have used imaging windows ranging from 4 to 9 cm [28–32]. Thus, the window depths were set at 5.0, 6.5, and 8.0 cm. The foci locations were set from 1.0 to 1.5, 2.5-3.0, and 4.0-4.5 cm to determine the effects of superficial, mid-belly, and deep focus locations, respectively. Finally, the gain was set at 46, 48, and 50%, which was determined based upon preliminary testing to provide images with enough contrast for fascicular visualization but without significant saturation [15, 29, 31]. The ultrasound machine used reports gain in % instead of dB. Representative B-mode images from 5 cm window depth are shown in Fig. 2. A complete set of B-mode images across all system levels used in the study can be found in the Supplementary Materials.

### Image analysis

**Fig. 3:**
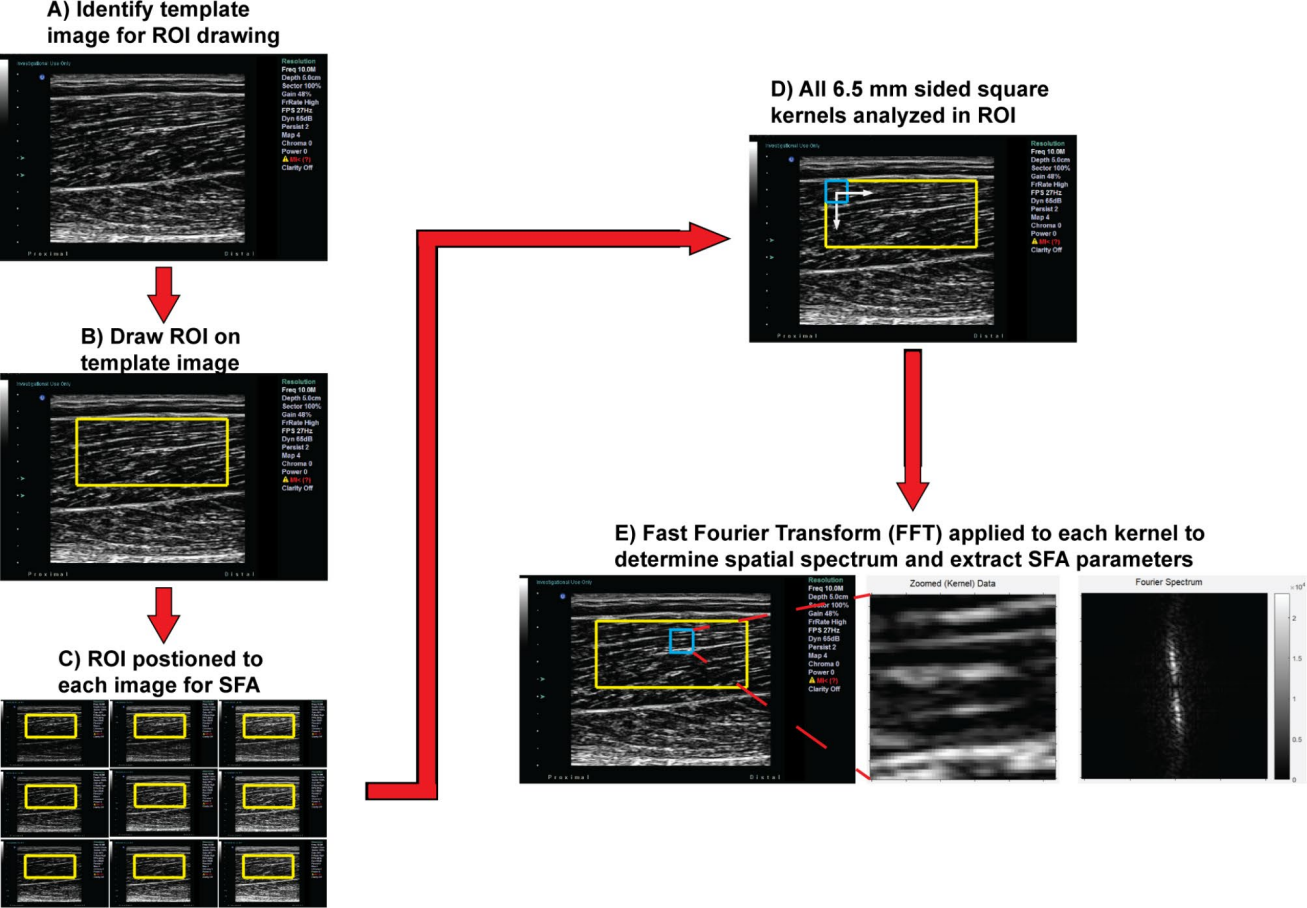
Data analysis procedure for extracting spatial frequency analysis (SFA) parameters across images. **(A)** Once all ultrasound images were collected, all images were reviewed to identify the image to be used as a template. **(B)** The parent region of interest (ROI) was then drawn on the template image. **(C)** A normalized two-dimensional cross correlation technique was used to ensure SFA parameters were extracted from the same ROI. **(D)** All kernels within the image were analyzed. **(E)** A Fast Fourier Transform (FFT) was applied to each kerenel and SFA parameters were extracted. Steps A-C were repeated for each window depth (5.0, 6.5, and 8.0 cm) and steps D and E were then performed for all image setting combinations across all participants

All longitudinal images captured from each participant were saved on the local computer as 800 × 600 pixel .png files for subsequent offline analysis. A flow chart of the entire analysis procedure is shown in Fig. 3, and all images were processed using custom MATLAB algorithms (Mathworks, Natick, MA). Although a previous investigation showed that the reliability of drawing a region of interest (ROI) from a single rater was excellent [8], a normalized two-dimensional cross-correlation technique was used between a template image and subsequent images for each trial for each participant. This method was utilized to minimize the amount of user-defined error in drawing the ROI across all images and to ensure spatial frequency parameters were extracted from the same region across all images. A rectangular ROI of the entire muscle thickness was drawn within one representative image of each window depth. The width of the ROI was limited to the middle portion of the image with each side approximately 0.5 cm from the lateral edges of the image to preserve lateral resolution (Fig. 3).

SFA parameter extraction was performed according to previously-established protocols [5, 8, 9]. Briefly, the spatial spectrum of each kernel was estimated by a Fast Fourier Transform (FFT) of the Hanning-weighted image kernel. The number of samples within each kernel was set to correspond to a square with 6.5 mm sides across all window depths and permitted to overlap in both the axial and lateral directions within the parent ROI. Kernels were zero-padded to 128 × 128 samples prior to FFT to increase frequency sampling. A 2D highpass filter in the frequency domain (-3 dB cut-off about 1.0 mm^− 1^) was also applied before parameter extraction to attenuate low spatial frequency artifacts [5].

Spatial frequency parameters were extracted for each kernel and averaged across the entire ROI. The spatial frequency parameters of interest were the peak spatial frequency radius (PSFR), Mmax, Sum, and Mmax%. The description and mathematical formulations of these parameters are detailed elsewhere [5, 9]. Briefly, the PSFR (the distance between the location of maximum amplitude and the origin) characterizes the most dominant spacing between the hyperechoic muscle fascicles. Mmax (the amplitude of the spatial frequency at the PSFR location) corresponds to the strength of the most prominent fascicular banded pattern. Sum (summation of the kernel pixels) corresponds to the image brightness at the kernel location. Mmax% is defined as the ratio of Mmax to Sum and is related to the most prominent fascicle pattern relative to the speckle background.

A custom semi-automated algorithm was developed to measure subcutaneous adipose thickness. This consisted of the user identifying and drawing a rectangular ROI around the dermis layer and the superficial aponeurosis. A binary mask was then created of the dermis and superficial aponeurosis and a second order polynomial line was fitted using the middle of the binary mask, which attempted to account for any slight curvature of either the dermis or aponeurosis. Using the same pixel normalization value used in determining the kernel size, the subcutaneous adipose thickness was calculated at the proximal (20%), middle (50%), and distal (80%) width of the image. The average of these three measures was then used for subsequent analysis.

### Statistical analyses

Separate linear mixed effects (LME) models were run for each SFA parameter to determine how the systematic changes in ultrasound system settings influenced the value of SFA parameters. Each LME model was adjusted for adipose thickness and had focus location, window depth, and gain input as fixed effects. Participants were input as random effects to account for the repeated measures within each participant. A full factorial model was initially implemented followed by removal of non-significant interaction terms. Post hoc Tukey tests were used to compare levels of significant main effects or interactions. The results are presented as least squares means [95% confidence interval] of the final LME models. The least squares mean (LSM) estimates for all parameters and levels are provided in the Supplementary Materials. All analyses were performed using R Studio [33] with *a priori* significance set at α = 0.05.

## Results

The demographics of participants are shown in Table 1. Due to equipment malfunction, the images were not saved for 4 participants resulting in the data analyses being performed on 36 participants. A total of 23 participants were randomly selected to participate in assessing the reliability of our measures which are reported in the Supplementary Materials.

**Table 1:** Participant demographics expressed as mean (standard deviation)

Characteristic	Male (n = 14)	Female (n = 22)	Total (n = 36)
Age (years)	22.9 (4.4)	24.1 (4.4)	23.6 (4.5)
Height (cm)	181.1 (5.6)	166.8 (5.3)	172.4 (8.8)
Weight (kg)	75.1 (8.8)	62.2 (5.9)	67.2 (9.6)
Body Mass Index (BMI) (kg/m^2^)	22.8 (1.8)	22.3 (1.5)	22.5 (1.6)
Subcutaneous Adipose Thickness (cm)	0.55 (0.17)	1.27 (0.33)	0.99 (0.45)

### Influence of machine settings on SFA parameters

#### Peak spatial frequency radius (PSFR)

**Fig. 4:**
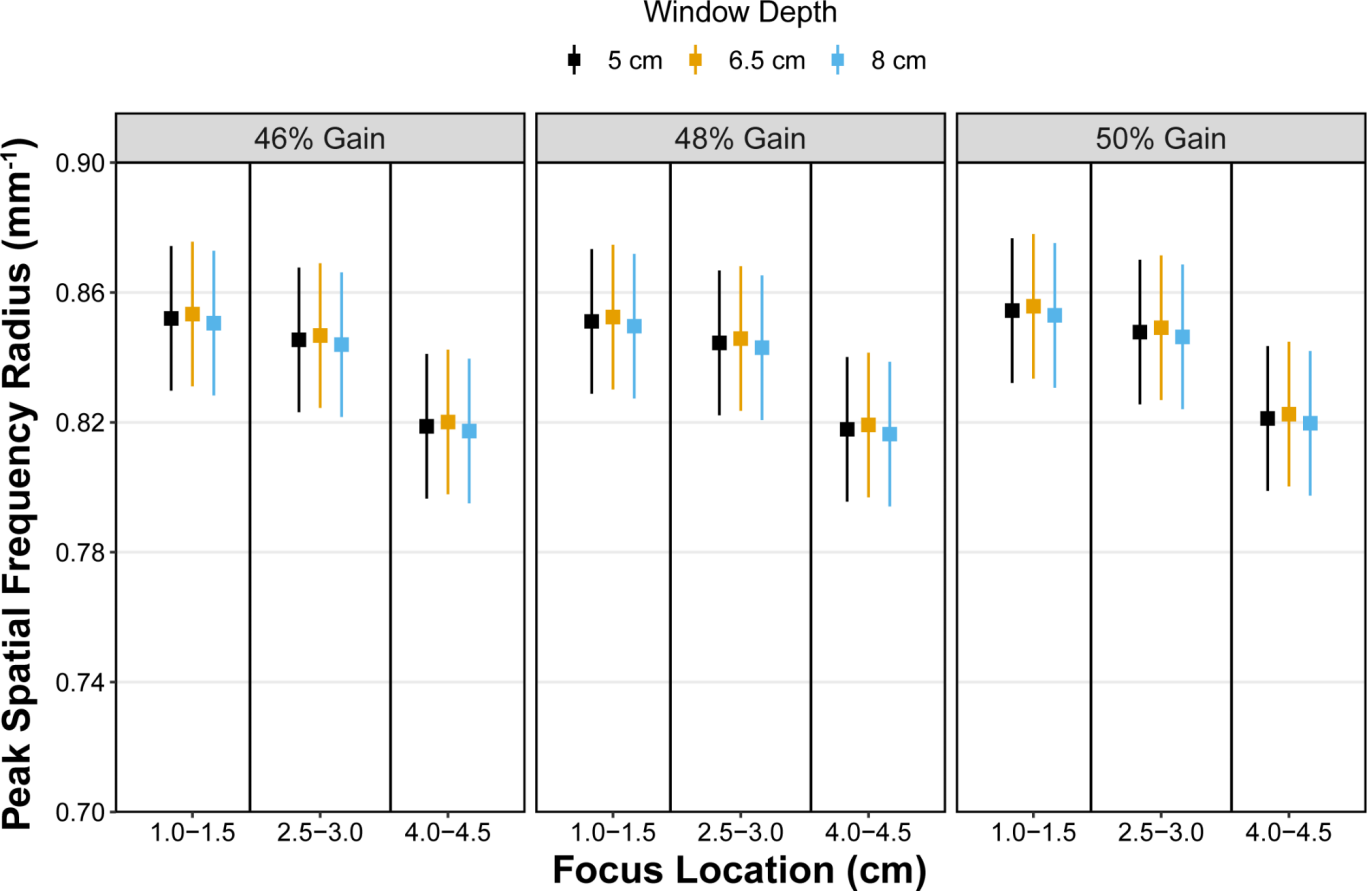
Least square mean estimates (squares) with 95% confidence intervals (whiskers) for peak spatial frequency radius. Estimates decreased only with respect to deeper focus locations

Least squares mean values for PSFR for all combinations of ultrasound settings are shown in Fig. 4. No significant interactions between any fixed effects were observed for PSFR. No associations between window depth (p = 0.48) and gain (p = 0.33) with PSFR were identified. The PSFR was significantly different between foci locations (p < 0.001) with differences noted between all pairwise comparisons. Specifically, PSFR was greater (p = 0.01) with a superficial (1.0-1.5 cm) focus location compared to a focus location at the mid-belly (2.5-3.0 cm) with a difference (standard error) of 0.007 (0.002) (LSM: 0.852 [0.830, 0.874] vs. LSM: 0.846 [0.824, 0.868], respectively). PSFR was also greater (p < 0.001) with a superficial location compared to a deep (4.0-4.5 cm) location with a difference of 0.03 (0.002) (LSM: 0.852 [0.830, 0.874] vs. 0.819 [0.797, 0.841], respectively). The PSFR was also greater with a mid-belly focus than a deep focus (p < 0.01) with a difference of 0.03 (0.002).

### Mmax

**Fig. 5:**
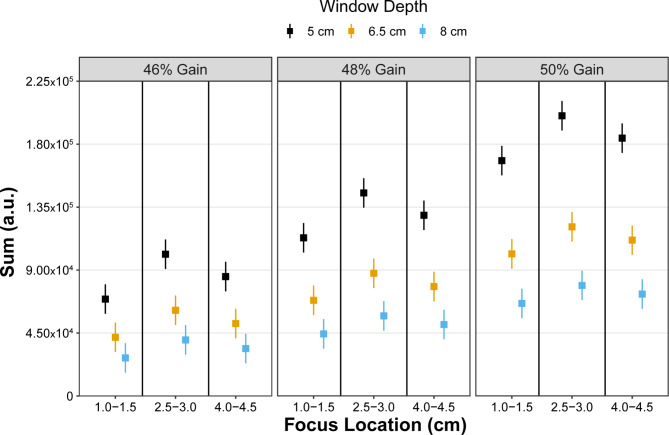
Least square mean estimates (squares) with 95% confidence intervals (whiskers) for Mmax. Estimates in Mmax were influenced in combination with increasing gain and decreasing window depth and in combination with increasing gain and focus location. a.u. = arbitrary units

Least squares mean values for Mmax for all combinations of ultrasound settings are shown in Fig. 5. Significant interactions between focus location*window depth (p < 0.001) and between gain*window depth (p < 0.001) were identified. Significant differences (p < 0.001) were identified between all pairwise comparisons of focus location and window depth except between superficial and deep locations at each window depth setting (p > 0.74). Post hoc pairwise comparisons showed significant differences (p < 0.001) between all window depth and gain comparisons except between 6.5 cm depth at 46% gain and 8 cm depth at 50% gain (p = 0.44). Estimates of Mmax for all pairwise comparisons for focus location*window depth and window depth*gain are additionally provided in the Supplementary Materials.

### Sum

**Fig. 6:**
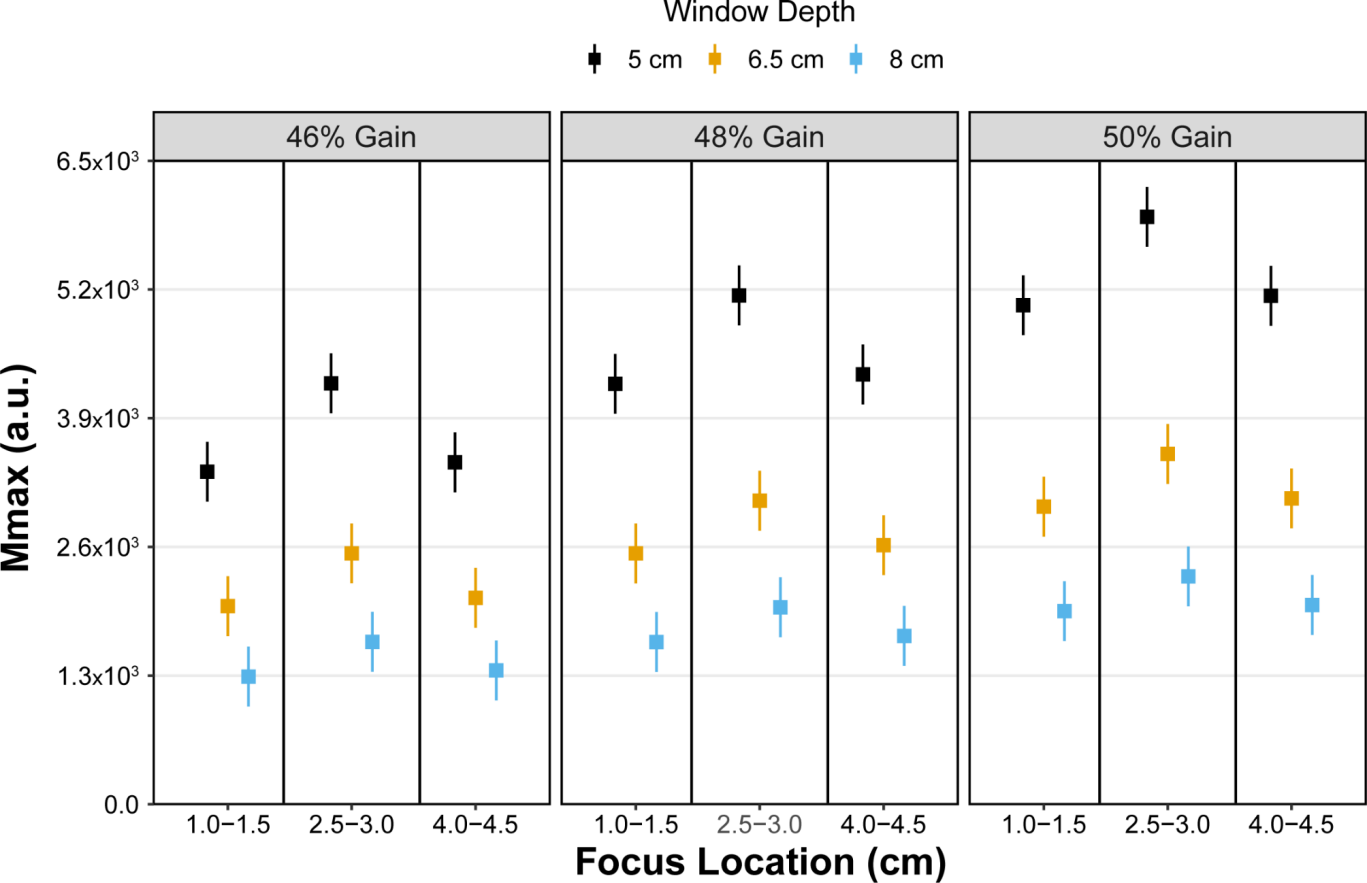
Least square mean estimates (squares) with 95% confidence intervals (whiskers) for Sum. Estimates in Sum were influenced in combination with increasing gain and decreasing window depth and in combination with increasing gain and focus location. a.u. = arbitrary units

Least squares mean values for Sum for all combinations of ultrasound settings are shown in Fig. 6. Significant interactions between focus location*window depth (p < 0.0001) and between gain*window depth (p < 0.0001) were identified for Sum. Significant differences were identified between all pairwise comparisons between focus locations and window depth (p < 0.04) with the exception between the mid-belly and deep focus locations at a window depth of 8 cm (p = 0.08). All pairwise comparisons between gain and window depth were significant (p < 0.02) except for 46% gain at 6.5 cm window depth and 48% gain at 8.0 cm depth (p = 0.99) and 48% gain at 6.5 cm depth and 50% gain at 8.0 cm depth (p = 0.19). Estimates of Sum values for all pairwise comparisons for focus location*window depth and window depth*gain are provided in the Supplementary Materials.

### Mmax%

**Fig. 7:**
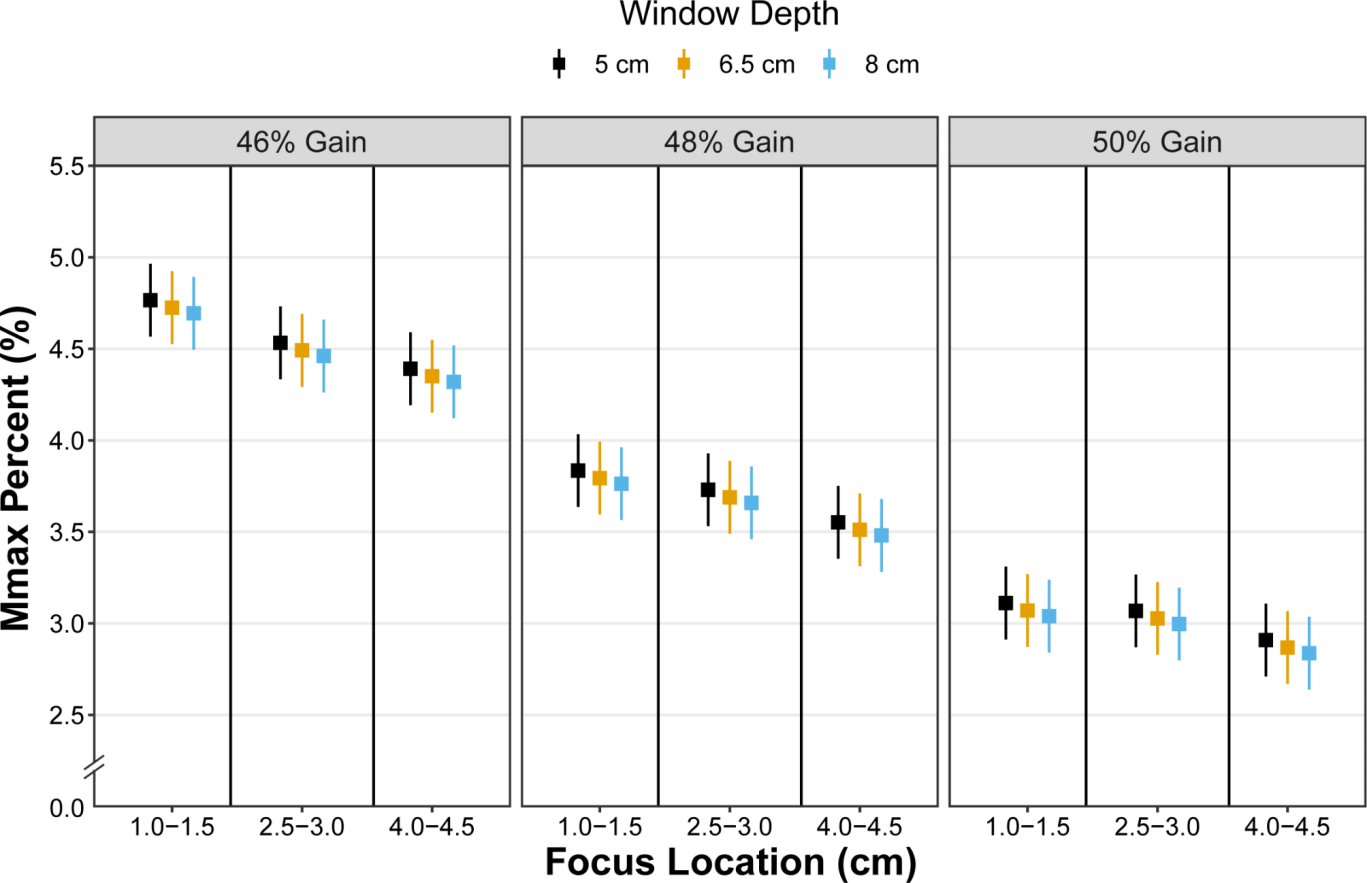
Least square mean estimates (squares) with 95% confidence intervals (whiskers) for Mmax%. Estimates decreased with increasing window depth and in combination with increasing gain and focus location

Least squares mean values for Mmax% for all combinations of ultrasound settings are shown in Fig. 7. A significant interaction was identified between gain*focus location for Mmax% (p < 0.001). No other two-way interactions were significant. Significant differences (p < 0.007) were identified between all pairwise comparisons except between superficial and deep focus locations at 50% gain (p = 0.85). Window depth was also associated with Mmax% (p < 0.001) with significant differences (p = 0.03) noted between 5.0 and 6.5 cm (LSM: 3.77 [3.57, 3.96] vs. 3.73 [3.53, 3.92], respectively; difference (standard error) between levels: 0.04 (0.02)) and 5.0 and 8.0 cm depths (LSM: 3.77 [3.57, 3.96] vs. 3.69 [3.50, 3.89], respectively; difference between levels: 0.07 (0.02), p < 0.001). No differences were identified between 6.5 and 8.0 cm window depths (p = 0.15). Estimates of Mmax% values for all pairwise comparisons of Mmax% estimates for focus location*gain are provided in the Supplementary Materials.

## Discussion

This work investigated the effects of ultrasound machine system settings on extracted SFA parameters in the biceps femoris muscle. We observed minimal changes in PSFR across machine settings, while Mmax and Sum were most sensitive to changes in machine settings consistent with our hypothesis. Mmax% was influenced by both gain and focus location and independently with window depth in contrast to our initial hypothesis.

The PSFR parameter is the most frequently reported SFA parameter in various studies investigating tendon structure and its relationship to clinical symptoms of tendinopathy [5–7]. This parameter is defined as the rotationally invariant distance from the origin to the spatial frequency with maximum amplitude in the 2-D Fourier spectrum. The PSFR corresponds to the most dominant spacing between hyperechoic fascicles within both tendon and muscle [5, 8, 9]. We observed significant differences in PSFR between each focus location. The lower PSFR for the deep focus location could possibly be explained by the fact that the parent ROI from which SFA parameters were extracted was located superficial to the deep focus location and corresponded approximately to the mid-belly focus location for most subjects. This would indicate that the lateral resolution in the ROI was highest (worst) for the deep focus location, perhaps bringing distinguishable features of the muscle (i.e., hyperechoic fascicles) out of focus. This finding is also consistent with early (non-SFA) studies showing minimal changes in texture features with ROI location [16]. It should be noted that in clinical imaging, the transmit focus location would be placed at approximately the level of the structure(s) of interest [15], so positioning the focus location below the ROI from which SFA parameters are extracted may not be meaningful clinically. Despite the observed differences in PSFR between focus locations, the differences (LSM < 0.03 mm^− 1^) are well below the minimal detectable change (MDC) previously determined in the biceps femoris muscle (MDC = $1.96*\sqrt{2}*0.05 {mm}^{-1}=0.14 {mm}^{-1}$) [8, 10]. Together these findings suggest that PSFR values are suitable for comparisons across individuals regardless of small adjustments in image acquisition settings since they appear to not be strongly tied to changes in gain and imaging depth.

Consistent with our initial hypothesis, both Mmax and Sum parameters were more affected by changes in ultrasound machine settings with both the interaction of focus location and window depth as well as window depth and gain influencing the extracted values. Mmax is the maximum amplitude of the 2D frequency spectrum, while Sum is the sum of frequency amplitudes within the spectrum corresponding to the overall image brightness [5, 8, 10]. Acoustic waves are more attenuated with deeper imaging, causing lower reflection with deep tissue reflectors (i.e., hyperechoic perimysium). As a result, both the maximum amplitude (Mmax) and total image brightness (Sum) would be expected to decrease with deeper imaging depths. In contrast, increased signal levels, which are manually manipulated by the sonographer by increasing the gain, would increase both parameters. Furthermore, the focus location would increase both the lateral resolution and the energy of the sound beams at the area of tissue where the focus is positioned. The parent ROI from which SFA parameters were extracted was positioned in the middle of the muscle belly, and for most individuals in this study, the parent ROI position ranged from 1.25 to 4 cm. As a result, both the superficial and deep focus locations were positioned near the ROI borders. This could explain the parabolic nature of both the Sum and Mmax values which peaked at the mid-belly location (where the lateral resolution and the energy of the sound beams were at a maximum for most individuals) across all window depths and gains (Figs. 5 and 6). Considering the sensitivity of these two parameters with respect to small modifications in ultrasound settings, care should be used when interpreting the values across studies or even across different time points within the same person if the settings are not consistent between imaging sessions.

Mmax% is another SFA parameter originally proposed [5]. This parameter is mathematically defined as the ratio of the maximum amplitude of the 2D frequency spectrum to the sum of frequency amplitudes within the spectrum. With respect to the muscle structure, Mmax% represents the strength of the most prominent hyperechoic striation pattern reflected by the fascicles relative to the overall image brightness [8, 10]. The combination of focus location and gain had a significant effect on the value of Mmax% with differences noted across most pairwise comparisons. These observations are consistent with basic ultrasound physics in that deeper focus locations reduce the clarity and prominence of the banded pattern superficial to the focus location. Increasing the gain results in increased image brightness, which is inversely related to Mmax% [9]. We also observed that Mmax% was independently associated with window depth with resultant decreases in Mmax% with increased window depth. This is likely due to greater attenuation of the sound waves with deeper imaging depths. Considering how this parameter is calculated, a deeper imaging window would reduce both the overall image brightness and the strength of the banded pattern as more energy is dissipated with both factors resulting in reduced Mmax% values. However, it should be noted that changes in window depth (with all other settings held constant) spanned differences of 0.07% which is well below the minimal detectable change of this parameter within the biceps femoris muscle (MDC = $1.96*\sqrt{2}*0.24\%=0.67\%$) [8]. These data suggest that if all other settings (i.e., gain and focus location) are consistent, then small modifications in imaging window depth may have minimal effect on Mmax%.

There are limitations to the study that should be considered when interpreting the results. Although we did have 36 participants included in the study for a total of 972 images analyzed, the participants were limited to individuals with a normal BMI. Considering the effect body habitus has on image quality with ultrasound imaging [18, 19], it is unclear if these findings would translate to other populations across a possibly wider range of ultrasound setting changes. Another limitation is that the study was conducted with only a single ultrasound machine and linear array transducer. Although we were only interested in changes of ultrasound settings from a preset value, previous studies have shown lower reliability in grayscale levels between different ultrasound machines [14], and it is unknown if the observed differences would be consistent across multiple ultrasound machines and/or transducer types. SFA parameters could also be influenced by changing other settings such as dynamic range or frequency. However, maintaining a constant dynamic range and probe frequency is consistent with previous quantitative ultrasound studies with the dynamic range used in this study (65 dB) falling in within typical ranges for muscle imaging (65–72 dB) [3, 34–37]. We did not investigate the effects of modifying the frequency of the transducer since it will, by definition, alter the parameters calculated by the frequency analysis methods used in this paper. Additionally, the highest frequency feasible to clearly visualize the tissue of interest (e.g., liver vs. Achilles tendon) is typically used clinically and modifying the frequency was outside the scope of this study. The extent or relationship (i.e., linear or nonlinear) of the changes in transducer frequency and SFA parameter values are not known, and future investigations may be warranted. Finally, this study only investigated the mid-belly of the biceps femoris muscle of the hamstrings. It is not clear if architectural variation along the muscle or between muscles [9, 38–40] would influence the sensitivity of the SFA parameters with changes in ultrasound settings. Future replication studies may attempt to determine if these observations would be similar across different ultrasound systems, transducer types, and different muscle groups.

## Conclusions

This study investigated the effects of small changes in ultrasound machine settings typically modified in clinical imaging on extracted spatial frequency parameters within the biceps femoris muscle. The PSFR was minimally affected by modifications in machine settings, suggesting this parameter is robust to small modifications for optimizing image quality in musculoskeletal ultrasound and may be well suited for comparison across individuals and between studies. Mmax and Sum parameters were sensitive to small changes in machine settings. Mmax% was influenced by gain and focus location but appears to be minimally affected by increasing the window depth. Based upon these observations, Mmax% may be a better alternative to Mmax and Sum in characterizing tissue structure if similar gain and focus settings are used within the same individual in bilateral imaging and in longitudinal studies. This work shows that frequently adjusted imaging parameters may be altered within typical limits without affecting PSFR, but care should be used when interpreting other parameters across studies or even across different time points within the same person if the settings are not consistent between imaging sessions. Taken together, it is recommended that when using SFA to characterize tissue structure and modifying ultrasound machine system settings around a preset, PSFR is the most robust to change and should be the primary parameter used. If investigators who use SFA desire to use another parameter to characterize tissue structure, Mmax% may be recommended. However, researchers should use caution in comparing Mmax% across studies if different imaging depths are used. Findings from this study may enable SFA to be used in other musculotendinous tissue studies, including muscle strain injury, where ultrasound settings are modified for optimal image quality.

### Electronic supplementary material

Below is the link to the electronic supplementary material.

Supplementary Material 1

Supplementary Material 2

Supplementary Material 3

Supplementary Material 4

## Data Availability

All data generated or analyzed during this study are included in this published article and its supplementary information files.

## Abbreviations

BMI: Body mass index
FFT: Fast Fourier Transform
LME: Linear mixed effects
MDC: Minimal detectable change
PSFR: Peak spatial frequency radius
ROI: Region of interest
SFA: Spatial frequency analysis
