# Supplementary material for: Influence of ultrasound machine settings on quantitative measures derived from spatial frequency analysis of muscle tissue
Source: BMC Musculoskelet Disord. 2023 Aug 22;24:664. doi: 10.1186/s12891-023-06790-3 (PMC10463672; doi:10.1186/s12891-023-06790-3)
Supplement: Supplementary file 1 — Supplementary Material 1 [file 12891_2023_6790_MOESM1_ESM.docx]

**Supplementary Materials**

**Table S1.** Least square mean (LS mean) estimates for peak spatial frequency radius (PSFR) across all ultrasound machine setting combinations.

| **Focus Location** | **Gain** | **Window Depth** | **LS Mean** | **Lower 95% Confidence Limit** | **Upper 95% Confidence Limit** |
| --- | --- | --- | --- | --- | --- |
| 1.0-1.5 cm | 46% | 5.0 cm | 0.852 | 0.830 | 0.874 |
| 2.5-3.0 cm | 46% | 5.0 cm | 0.845 | 0.823 | 0.868 |
| 4.0-4.5 cm | 46% | 5.0 cm | 0.819 | 0.797 | 0.841 |
| 1.0-1.5 cm | 48% | 5.0 cm | 0.851 | 0.829 | 0.873 |
| 2.5-3.0 cm | 48% | 5.0 cm | 0.844 | 0.822 | 0.867 |
| 4.0-4.5 cm | 48% | 5.0 cm | 0.818 | 0.796 | 0.840 |
| 1.0-1.5 cm | 50% | 5.0 cm | 0.854 | 0.832 | 0.877 |
| 2.5-3.0 cm | 50% | 5.0 cm | 0.848 | 0.826 | 0.870 |
| 4.0-4.5 cm | 50% | 5.0 cm | 0.821 | 0.799 | 0.843 |
| 1.0-1.5 cm | 46% | 6.5 cm | 0.853 | 0.831 | 0.876 |
| 2.5-3.0 cm | 46% | 6.5 cm | 0.847 | 0.824 | 0.869 |
| 4.0-4.5 cm | 46% | 6.5 cm | 0.820 | 0.798 | 0.842 |
| 1.0-1.5 cm | 48% | 6.5 cm | 0.852 | 0.830 | 0.875 |
| 2.5-3.0 cm | 48% | 6.5 cm | 0.846 | 0.824 | 0.868 |
| 4.0-4.5 cm | 48% | 6.5 cm | 0.819 | 0.797 | 0.841 |
| 1.0-1.5 cm | 50% | 6.5 cm | 0.856 | 0.833 | 0.878 |
| 2.5-3.0 cm | 50% | 6.5 cm | 0.849 | 0.827 | 0.871 |
| 4.0-4.5 cm | 50% | 6.5 cm | 0.823 | 0.800 | 0.845 |
| 1.0-1.5 cm | 46% | 8.0 cm | 0.851 | 0.828 | 0.873 |
| 2.5-3.0 cm | 46% | 8.0 cm | 0.844 | 0.822 | 0.866 |
| 4.0-4.5 cm | 46% | 8.0 cm | 0.817 | 0.795 | 0.840 |
| 1.0-1.5 cm | 48% | 8.0 cm | 0.850 | 0.827 | 0.872 |
| 2.5-3.0 cm | 48% | 8.0 cm | 0.843 | 0.821 | 0.865 |
| 4.0-4.5 cm | 48% | 8.0 cm | 0.816 | 0.794 | 0.839 |
| 1.0-1.5 cm | 50% | 8.0 cm | 0.853 | 0.831 | 0.875 |
| 2.5-3.0 cm | 50% | 8.0 cm | 0.846 | 0.824 | 0.869 |
| 4.0-4.5 cm | 50% | 8.0 cm | 0.820 | 0.797 | 0.842 |

^†^ Final linear mixed effects model: PSFR ~ AdiposeThickness + FocusLocation + WindowDepth + Gain + (1 | Subject)

**Table S2.** Least square mean (LS mean) estimates for Mmax across all ultrasound machine setting combinations.

| **Focus Location** | **Gain** | **Window Depth** | **LS Mean** | **Lower 95% Confidence Limit** | **Upper 95% Confidence Limit** |
| --- | --- | --- | --- | --- | --- |
| 1.0-1.5 cm | 46% | 5.0 cm | 3360 | 3045 | 3674 |
| 2.5-3.0 cm | 46% | 5.0 cm | 4245 | 3931 | 4560 |
| 4.0-4.5 cm | 46% | 5.0 cm | 3460 | 3146 | 3775 |
| 1.0-1.5 cm | 48% | 5.0 cm | 4249 | 3934 | 4563 |
| 2.5-3.0 cm | 48% | 5.0 cm | 5143 | 4829 | 5458 |
| 4.0-4.5 cm | 48% | 5.0 cm | 4338 | 4023 | 4652 |
| 1.0-1.5 cm | 50% | 5.0 cm | 5039 | 4725 | 5354 |
| 2.5-3.0 cm | 50% | 5.0 cm | 5938 | 5623 | 6252 |
| 4.0-4.5 cm | 50% | 5.0 cm | 5134 | 4820 | 5449 |
| 1.0-1.5 cm | 46% | 6.5 cm | 2001 | 1687 | 2316 |
| 2.5-3.0 cm | 46% | 6.5 cm | 2528 | 2213 | 2842 |
| 4.0-4.5 cm | 46% | 6.5 cm | 2093 | 1778 | 2407 |
| 1.0-1.5 cm | 48% | 6.5 cm | 2530 | 2215 | 2844 |
| 2.5-3.0 cm | 48% | 6.5 cm | 3070 | 2755 | 3384 |
| 4.0-4.5 cm | 48% | 6.5 cm | 2618 | 2304 | 2932 |
| 1.0-1.5 cm | 50% | 6.5 cm | 3011 | 2697 | 3325 |
| 2.5-3.0 cm | 50% | 6.5 cm | 3543 | 3228 | 3857 |
| 4.0-4.5 cm | 50% | 6.5 cm | 3082 | 2767 | 3396 |
| 1.0-1.5 cm | 46% | 8.0 cm | 1292 | 977 | 1606 |
| 2.5-3.0 cm | 46% | 8.0 cm | 1639 | 1325 | 1954 |
| 4.0-4.5 cm | 46% | 8.0 cm | 1352 | 1037 | 1666 |
| 1.0-1.5 cm | 48% | 8.0 cm | 1639 | 1325 | 1953 |
| 2.5-3.0 cm | 48% | 8.0 cm | 1991 | 1677 | 2306 |
| 4.0-4.5 cm | 48% | 8.0 cm | 1700 | 1385 | 2014 |
| 1.0-1.5 cm | 50% | 8.0 cm | 1950 | 1635 | 2264 |
| 2.5-3.0 cm | 50% | 8.0 cm | 2302 | 1988 | 2616 |
| 4.0-4.5 cm | 50% | 8.0 cm | 2015 | 1700 | 2329 |

^†^ Final linear mixed effects model: Mmax ~ AdiposeThickness + FocusLocation + WindowDepth + Gain + FocusLocation*WindowDepth + Gain*WindowDepth + (1 | Subject)

**Table S3.** Mmax least square means estimates for combination of focus locations and window depths. Differences were identified between all pairwise comparisons except between pairs with the same letter.

| **Focus Location** | **Window Depth** | **Mmax Estimate**  **[95% Confidence Interval]** |
| --- | --- | --- |
| 1.0-1.5 cm | 5 cm | 4216 [3920, 4512]^a^ |
| 2.5-3.0 cm | 5 cm | 5109 [4813. 5405] |
| 4.0-4.5 cm | 5 cm | 4311 [4015, 4607]^a^ |
| 1.0-1.5 cm | 6.5 cm | 2514 [2218, 2810]^b^ |
| 2.5-3.0 cm | 6.5 cm | 3047 [2751, 3343] |
| 4.0-4.5 cm | 6.5 cm | 2597 [2301, 2894]^b^ |
| 1.0-1.5 cm | 8 cm | 1627 [1331, 1923]^c^ |
| 2.5-3.0 cm | 8 cm | 1978 [1681, 2274] |
| 4.0-4.5 cm | 8 cm | 1689 [1393, 1985]^c^ |

**Table S4.** Mmax least square means estimates for combination of window depths and gain. Differences were identified between all pairwise comparisons except between pairs with the same letter.

| **Window Depth** | **Gain** | **Mmax Estimate**  **[95% Confidence Interval]** |
| --- | --- | --- |
| 5 cm | 46% | 3689 [3392, 3985] |
| 6.5 cm | 46% | 2207 [1911, 2503]^a^ |
| 8 cm | 46% | 1428 [1131, 1724] |
| 5 cm | 48% | 4577 [4280, 4873] |
| 6.5 cm | 48% | 2739 [2443, 3035] |
| 8 cm | 48% | 1777 [1481, 2073] |
| 5 cm | 50% | 5370 [5074, 5667] |
| 6.5 cm | 50% | 3212 [2916, 3508] |
| 8 cm | 50% | 2089 [1793, 2385]^a^ |

**Table S5.** Least square mean (lsmean) estimates for Sum across all ultrasound machine setting combinations.

| **Focus Location** | **Gain** | **Window Depth** | **LS Mean** | **Lower 95% Confidence Limit** | **Upper 95% Confidence Limit** |
| --- | --- | --- | --- | --- | --- |
| 1.0-1.5 cm | 46% | 5.0 cm | 69263 | 58717 | 79809 |
| 2.5-3.0 cm | 46% | 5.0 cm | 101354 | 90808 | 111900 |
| 4.0-4.5 cm | 46% | 5.0 cm | 85359 | 74813 | 95905 |
| 1.0-1.5 cm | 48% | 5.0 cm | 113084 | 102537 | 123630 |
| 2.5-3.0 cm | 48% | 5.0 cm | 145175 | 134628 | 155721 |
| 4.0-4.5 cm | 48% | 5.0 cm | 129180 | 118633 | 139726 |
| 1.0-1.5 cm | 50% | 5.0 cm | 168232 | 157686 | 178779 |
| 2.5-3.0 cm | 50% | 5.0 cm | 200323 | 189777 | 210870 |
| 4.0-4.5 cm | 50% | 5.0 cm | 184329 | 173782 | 194875 |
| 1.0-1.5 cm | 46% | 6.5 cm | 41944 | 31397 | 52491 |
| 2.5-3.0 cm | 46% | 6.5 cm | 61272 | 50726 | 71819 |
| 4.0-4.5 cm | 46% | 6.5 cm | 51794 | 41247 | 62340 |
| 1.0-1.5 cm | 48% | 6.5 cm | 68396 | 57850 | 78942 |
| 2.5-3.0 cm | 48% | 6.5 cm | 87724 | 77178 | 98270 |
| 4.0-4.5 cm | 48% | 6.5 cm | 78245 | 67699 | 88792 |
| 1.0-1.5 cm | 50% | 6.5 cm | 101590 | 91044 | 112136 |
| 2.5-3.0 cm | 50% | 6.5 cm | 120919 | 110371 | 131466 |
| 4.0-4.5 cm | 50% | 6.5 cm | 111440 | 100893 | 121987 |
| 1.0-1.5 cm | 46% | 8.0 cm | 27231 | 16684 | 37779 |
| 2.5-3.0 cm | 46% | 8.0 cm | 40081 | 29533 | 50628 |
| 4.0-4.5 cm | 46% | 8.0 cm | 33918 | 23370 | 44466 |
| 1.0-1.5 cm | 48% | 8.0 cm | 44432 | 33885 | 54978 |
| 2.5-3.0 cm | 48% | 8.0 cm | 57281 | 46735 | 67827 |
| 4.0-4.5 cm | 48% | 8.0 cm | 51118 | 40572 | 61665 |
| 1.0-1.5 cm | 50% | 8.0 cm | 66174 | 55627 | 76720 |
| 2.5-3.0 cm | 50% | 8.0 cm | 79023 | 68477 | 89569 |
| 4.0-4.5 cm | 50% | 8.0 cm | 72860 | 62314 | 83406 |

^†^ Final linear mixed effects model: Sum~AdiposeThickness + FocusLocation + Gain + WindowDepth + Gain*WindowDepth + FocusLocation*WindowDepth + (1|SubjectID)

**Table S6.** Sum least square means estimates for combination of focus locations and window depths. Differences were identified between all pairwise comparisons except between pairs with the same letter.

| **Focus Location** | **Window Depth** | **Sum Estimate**  **[95% Confidence Interval]** |
| --- | --- | --- |
| 1.0-1.5 cm | 5 cm | 116860 [106568, 127151] |
| 2.5-3.0 cm | 5 cm | 148951 [138659, 159242] |
| 4.0-4.5 cm | 5 cm | 132956 [122664, 143248] |
| 1.0-1.5 cm | 6.5 cm | 70643 [60352, 80935] |
| 2.5-3.0 cm | 6.5 cm | 89972 [79680, 100263] |
| 4.0-4.5 cm | 6.5 cm | 80493 [70201, 90785] |
| 1.0-1.5 cm | 8 cm | 45946 [35654, 56237] |
| 2.5-3.0 cm | 8 cm | 58795 [48503, 69086] ^a^ |
| 4.0-4.5 cm | 8 cm | 52632 [42340, 62924] ^a^ |

**Table S7.** Sum least square means estimates for combination of window depths and gain. Differences were identified between all pairwise comparisons except between pairs with the same letter.

| **Gain** | **Window Depth** | **Sum Estimate**  **[95% Confidence Interval]** |
| --- | --- | --- |
| 46% | 5 cm | 85325 [75034, 95617] |
| 48% | 5 cm | 129146 [118854, 139438] |
| 50% | 5 cm | 184295 [174003, 194587] |
| 46% | 6.5 cm | 51670 [41378, 61962]^a^ |
| 48% | 6.5 cm | 78122 [67830, 88413]^b^ |
| 50% | 6.5 cm | 111316 [101024, 121608] |
| 46% | 8 cm | 33743 [23450, 44036] |
| 48% | 8 cm | 50944 [40652, 61235]^a^ |
| 50% | 8 cm | 72686 [62394, 82977]^b^ |

**Table S8.** Least square mean (lsmean) estimates for Mmax% across all ultrasound machine setting combinations.

| **Focus Location** | **Gain** | **Window Depth** | **LS Mean** | **Lower 95% Confidence Limit** | **Upper 95% Confidence Limit** |
| --- | --- | --- | --- | --- | --- |
| 1.0-1.5 cm | 46% | 5.0 cm | 4.766 | 4.567 | 4.965 |
| 2.5-3.0 cm | 46% | 5.0 cm | 4.532 | 4.334 | 4.731 |
| 4.0-4.5 cm | 46% | 5.0 cm | 4.391 | 4.192 | 4.590 |
| 1.0-1.5 cm | 48% | 5.0 cm | 3.835 | 3.636 | 4.034 |
| 2.5-3.0 cm | 48% | 5.0 cm | 3.730 | 3.531 | 3.929 |
| 4.0-4.5 cm | 48% | 5.0 cm | 3.552 | 3.353 | 3.751 |
| 1.0-1.5 cm | 50% | 5.0 cm | 3.111 | 2.913 | 3.310 |
| 2.5-3.0 cm | 50% | 5.0 cm | 3.068 | 2.870 | 3.267 |
| 4.0-4.5 cm | 50% | 5.0 cm | 2.909 | 2.710 | 3.108 |
| 1.0-1.5 cm | 46% | 6.5 cm | 4.725 | 4.526 | 4.924 |
| 2.5-3.0 cm | 46% | 6.5 cm | 4.492 | 4.293 | 4.690 |
| 4.0-4.5 cm | 46% | 6.5 cm | 4.350 | 4.151 | 4.549 |
| 1.0-1.5 cm | 48% | 6.5 cm | 3.794 | 3.595 | 3.993 |
| 2.5-3.0 cm | 48% | 6.5 cm | 3.689 | 3.490 | 3.888 |
| 4.0-4.5 cm | 48% | 6.5 cm | 3.511 | 3.313 | 3.710 |
| 1.0-1.5 cm | 50% | 6.5 cm | 3.071 | 2.872 | 3.269 |
| 2.5-3.0 cm | 50% | 6.5 cm | 3.027 | 2.829 | 3.226 |
| 4.0-4.5 cm | 50% | 6.5 cm | 2.868 | 2.670 | 3.067 |
| 1.0-1.5 cm | 46% | 8.0 cm | 4.694 | 4.495 | 4.893 |
| 2.5-3.0 cm | 46% | 8.0 cm | 4.461 | 4.262 | 4.660 |
| 4.0-4.5 cm | 46% | 8.0 cm | 4.319 | 4.121 | 4.518 |
| 1.0-1.5 cm | 48% | 8.0 cm | 3.763 | 3.565 | 3.962 |
| 2.5-3.0 cm | 48% | 8.0 cm | 3.658 | 3.460 | 3.857 |
| 4.0-4.5 cm | 48% | 8.0 cm | 3.481 | 3.282 | 3.679 |
| 1.0-1.5 cm | 50% | 8.0 cm | 3.040 | 2.841 | 3.239 |
| 2.5-3.0 cm | 50% | 8.0 cm | 2.997 | 2.798 | 3.195 |
| 4.0-4.5 cm | 50% | 8.0 cm | 2.838 | 2.639 | 3.036 |

^†^ Final linear mixed effects model: MmaxPercent~AdiposeThickness + FocusLocation + Gain + WindowDepth + Gain*FocusLocation + (1|SubjectID)

**Table S9.** Mmax% least square means estimates for combination of focus location and gain. Differences were identified between all pairwise comparisons except between pairs with the same letter.

| **Focus Location** | **Gain** | **Mmax% Estimate**  **[95% Confidence Interval]** |
| --- | --- | --- |
| 1.0-1.5 cm | 46% | 4.73 [4.53, 4.93] |
| 1.0-1.5 cm | 48% | 3.80 [3.60, 4.00] |
| 1.0-1.5 cm | 50% | 3.07 [2.88, 3.27]^a^ |
| 2.5-3.0 cm | 46% | 4.49 [4.30, 4.69] |
| 2.5-3.0 cm | 48% | 3.69 [3.49, 3.89] |
| 2.5-3.0 cm | 50% | 3.03 [2.83, 3.23]^a^ |
| 4.0-4.5 cm | 46% | 4.35 [4.16, 4.55] |
| 4.0-4.5 cm | 48% | 3.51 [3.32, 3.71] |
| 4.0-4.5 cm | 50% | 2.87 [2.67, 3.07] |
