# Supplementary material for: Influence of ultrasound machine settings on quantitative measures derived from spatial frequency analysis of muscle tissue
Source: BMC Musculoskelet Disord. 2023 Aug 22;24:664. doi: 10.1186/s12891-023-06790-3 (PMC10463672; doi:10.1186/s12891-023-06790-3)
Supplement: Supplementary file 2 — Supplementary Material 2 [file 12891_2023_6790_MOESM2_ESM.docx]

*Reliability of Image Analysis and Spatial Frequency Analysis Parameters*

Reliability was determined in both intra-session and inter-session procedures. In order to assess the repeatability and reliability of the procedures, an *a priori* power analysis using RStudio statistical software was performed. A sample size of 23 participants was calculated to determine the reliability of these methods (based upon an intraclass correlation coefficient (ICC) hypothesized value of 0.90, a reference ICC value of 0.70, 2 raters, a significance value of 0.05, and power of 0.80). Participants were randomized prior to study initiation to determine individuals who would undergo the reliability procedures.

For the intra-session reliability, the participant lied prone on the exam table in a relaxed state with their hips and knees in a neutral position and their feet suspended off the end of the exam table. After the initial image acquisition, the transducer was removed from the participant and then replaced in the same location to determine the intra-session reliability [1, 2]. To determine the inter-session reliability, the mid-thigh length mark was erased from the previous image acquisition. Following a 20 minute rest, the participant was re-positioned on the exam table, thigh length re-measured, and the identical imaging procedures were performed. A total of 81 images (27 per trial) were acquired for each of the 23 reliability participants.

*Statistical Analyses*

Intraclass correlation coefficients (ICC (3,1)) were calculated according to Koo et al. [3] to determine intra- and inter-session reliability. ICCs [95% confidence intervals] were determined using the *icc* function in the ‘irr’ package in R statistical software. Reliability was defined as poor (ICC < 0.5), moderate (0.5 < ICC < 0.75), good (0.75 < ICC < 0.9), or excellent (ICC > 0.9) for both intra-session and inter-session reliability measures. The coefficient of variation (CV) was calculated for each participant across all 27 images of each trial and for each of the 3 trials. The CV means and SD calculated across all 23 participants are reported. The standard error of measure (SEM) was calculated as $SEM= SD\sqrt{1-ICC}$ [4]. The minimal detectable change (MDC) was calculated as $MDC=SEM\times1.96 \times\sqrt{2}$.

**Table 1.** Coefficient of Variation of Adipose Thickness Measures and Spatial Frequency Analysis Parameters.

| **Variable** | **Trial 1 (%)** | **Trial 2 (%)** | **Trial 3 (%)** | **Overall (%)** |
| --- | --- | --- | --- | --- |
| Adipose Thickness (cm) | 1.1 (1.5) | 1.2 (1.7) | 1.2 (1.7) | 1.2 (1.6) |
| PSFR (mm^-1^) | 4.1 (2.1) | 3.5 (1.9) | 4.1 (2.2) | 3.9 (2.1) |
| Mmax (a.u.) | 15.8 (6.9) | 16.0 (6.0) | 15.2 (6.5) | 15.7 (6.5) |
| Sum (a.u.) | 16.8 (8.1) | 16.4 (7.3) | 16.2 (7.95) | 16.5 (7.8) |
| Mmax% (%) | 5.3 (2.6) | 5.6 (2.9) | 5.1 (2.6) | 5.3 (2.7) |

* Coefficient of variation (CV) was calculated across the three images for each focus location, and gain setting by each participant’s trials. The CV was then averaged across each trial for each variable and an overall CV was calculated across all trials.

**Table 2.** Intraclass Correlation Coefficients (ICC), Standard of Error of Measure (SEM), and Minimal Detectable Change (MDC) of Spatial Frequency Analysis Parameters and Adipose Thickness Measures.

| **Variable** | **Intra-Session** | | | **Inter-Session** | | |
| --- | --- | --- | --- | --- | --- | --- |
|  | **ICC(3,1)** | **SEM** | **MDC** | **ICC(3,1)** | **SEM** | **MDC** |
| Adipose Thickness (cm) | 1.00 [0.99, 1.00] | 0.03 | 0.08 | 0.99 [0.98, 0.99] | 0.06 | 0.17 |
| PSFR (mm^-1^) | 0.76 [0.74, 0.78] | 0.04 | 0.12 | 0.67 [0.64, 0.70] | 0.05 | 0.13 |
| Mmax (a.u.) | 0.94 [0.93, 0.95] | 374 | 1,038 | 0.94 [0.92, 0.95] | 397 | 1,100 |
| Sum (a.u.) | 0.97 [0.97, 0.98] | 9,497 | 26,323 | 0.98 [0.98, 0.98] | 8,641 | 23,950 |
| Mmax% (%) | 0.94 [0.93, 0.95] | 0.26 | 0.72 | 0.90 [0.83, 0.93] | 0.35 | 0.98 |

^†^ICC(3,1) calculated as two-way mixed, absolute agreement, single rater and expressed as ICC [95% confidence interval].

**References**

1. Freitas SR, Marmeleira J, Valamatos MJ, Blazevich A, Mil-Homens P. Ultrasonographic Measurement of the Biceps Femoris Long-Head Muscle Architecture. J Ultrasound Med. 2018;37:977–86.

2. Pimenta R, Blazevich AJ, Freitas SR. Biceps femoris long-head architecture assessed using different sonographic techniques. Med Sci Sports Exerc. 2018;50:2584–94.

3. Koo TK, Li MY. A Guideline of Selecting and Reporting Intraclass Correlation Coefficients for Reliability Research. J Chiropr Med. 2016;15:155–63.

4. Weir JP. Quantifying test-retest reliability using the intraclass correlation coefficient and the SEM. J Strength Cond Res. 2005;19:231–40.
