# Supplementary material for: Influence of ultrasound machine settings on quantitative measures derived from spatial frequency analysis of muscle tissue
Source: BMC Musculoskelet Disord. 2023 Aug 22;24:664. doi: 10.1186/s12891-023-06790-3 (PMC10463672; doi:10.1186/s12891-023-06790-3)
Supplement: Supplementary file 3 — Supplementary Material 3 [file 12891_2023_6790_MOESM3_ESM.docx]

*Normalized Cross-Correlation Summary Statistics*

The absolute proximo-distal and superficial-deep pixel displacements from the normalized two-dimensional cross-correlation technique are described in Table 1 below. The calibration factor is given to provide the approximate spatial displacements of the regions of interest between the template image and each subsequent image of the cross-correlation method.

The correlation coefficients from the normalized two-dimensional cross-correlation method are shown in Figure 2. The median (first quartile, third quartile) correlation coefficient across all images was r = 0.82 (0.76, 0.94). The medians by window depth are shown in Figure 2.

**Table 1.** Pixel Displacements from Region of Interest Drawn on Template Image and Each Subsequent Image for Spatial Frequency Analysis. Data are presented as median (minimum, maximum).

| **Window Depth** | **Proximo-Distal Displacement (pixels)** | **Superficial-Deep Displacement (pixels)** | **Calibration Factor (mm/pixel)** |
| --- | --- | --- | --- |
| 5.0 cm | 1.5 (0, 8) | 0 (0, 6) | 8 |
| 6.5 cm | 1.0 (0, 13) | 0 (0, 5) | 6 |
| 8.0 cm | 2.5 (1, 8) | 0 (0, 6) | 5 |


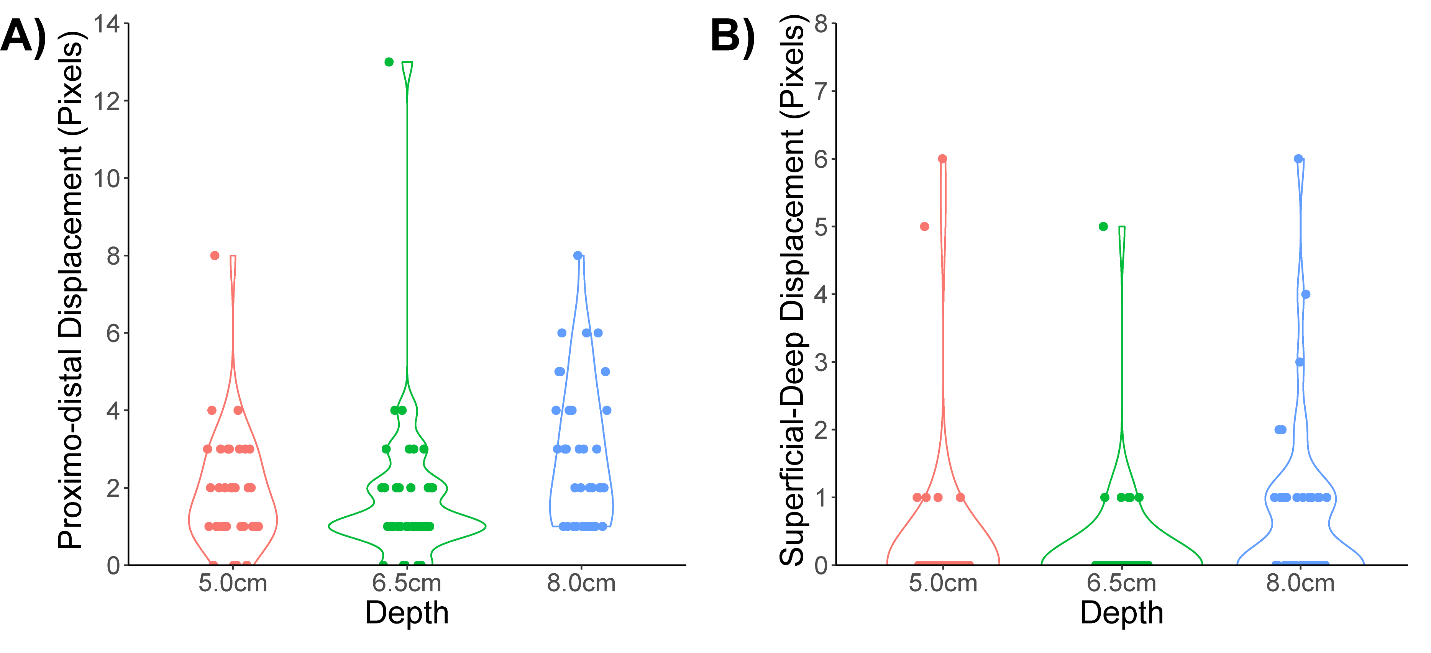


**Figure 1.** Pixel Displacements from Template Image Region of Interest and Region of Interest Placement Within Each Subsequent Image. A) Proximo-distal (left-right) pixel displacements shown by window depth across all participants. B) Superficial-deep (top-bottom) pixel displacements shown by window depth across all participants.


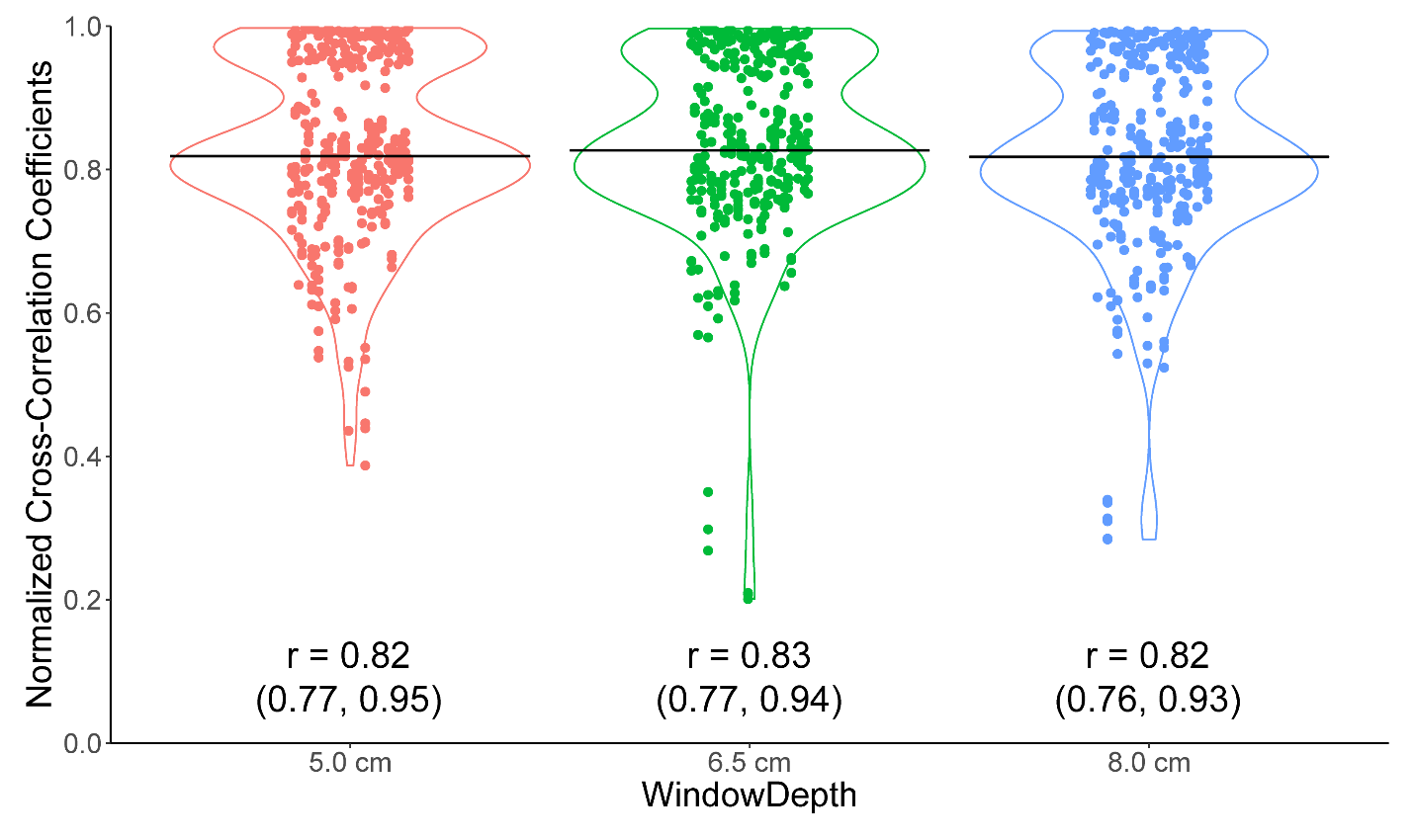


**Figure 2.** Correlation Coefficients from Two-Dimensional Cross Correlation Method for Region of Interest Placement Across All Images by Window Depth. Median correlation coefficients are given as median (first quartile, third quartile).
