# Supplementary figures and images for: Influence of ultrasound machine settings on quantitative measures derived from spatial frequency analysis of muscle tissue
Source: BMC Musculoskelet Disord. 2023 Aug 22;24:664. doi: 10.1186/s12891-023-06790-3 (PMC10463672; doi:10.1186/s12891-023-06790-3)

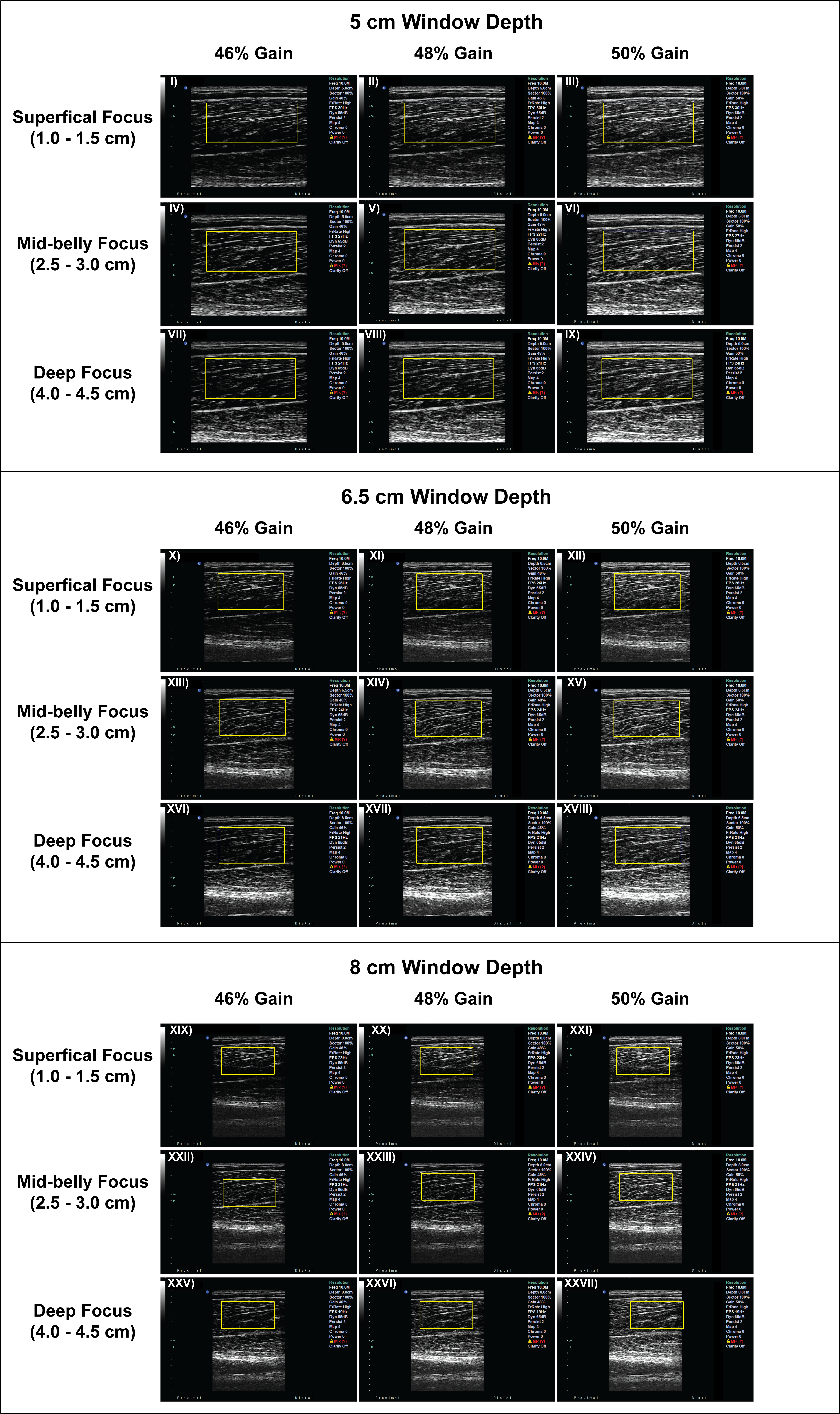

Supplement: Supplementary file 4 — Supplementary Material 4 [file 12891_2023_6790_MOESM4_ESM.tif]
